# Fuzzy adaptive fault diagnosis and compensation for variable structure hypersonic vehicle with multiple faults

**DOI:** 10.1371/journal.pone.0256200

**Published:** 2021-08-13

**Authors:** Kaiyu Hu, Wenhao Li, Zian Cheng

**Affiliations:** 1 Flight Control Research Institute, Nanjing University of Aeronautics and Astronautics, Nanjing, Jiangsu, China; 2 College of Automation Engineering, Nanjing University of Aeronautics and Astronautics, Nanjing, Jiangsu, China; National Huaqiao University, CHINA

## Abstract

Based on the type-II fuzzy logic, this paper proposes a robust adaptive fault diagnosis and fault-tolerant control (FTC) scheme for multisensor faults in the variable structure hypersonic vehicles with parameter uncertainties. Type-II fuzzy method approximates the original models while eliminating the parameter uncertainties. Hence the sensor faults are detected and isolated by the multiple output residuals and thresholds considering nonlinear approximation errors and disturbance. Based on the fuzzy adaptive augmented observer, the faults and disturbance are all estimated accurately by an improved proportional-differential part. Then a variable structure FTC scheme repairs the faults by the estimation, the fast-varying disturbance is considered in FTC scheme and is compensated by the control parameters designed based on its derivative function, thereby enhancing the output robust tracking accuracy of the variable structure hypersonic vehicles. The Lyapunov theory proves the system robust stability, semi-physical simulation verifies the validity of the proposed method and the superiority compared with the traditional method.

## 1. Introduction

Advanced hypersonic flight vehicles (HFVs) are equipped with active variable-structure fuselages to reduce air resistance and provide redundant control torque [1–3]. However, variable-structure HFVs are more sensitive to the influence of external disturbances and uncertainties than other aircraft are; thus, they are required to be more robust [4, 5]. Furthermore, changes in atmospheric conditions usually lead to actuator and sensor faults, thereby causing a catastrophic effect on flight performance [6–8]. Accordingly, research on the fault-tolerant control (FTC) technology for variable-structure HFVs can improve the reliability of this type of aircraft under complex flight conditions and widen the applicability of HFVs.

In recent years, there has been considerable progress in research on HFV control systems, involving not only traditional control but also fault repair control [9, 10]. The traditional control under fault-free conditions is primarily dedicated to overcome interference, uncertainty, time lag, and misaligned response. Liu et al. presented a new output feedback control design for the robust velocity and altitude tracking of HFVs; the control scheme was based on the assumption that only partial states of the HFV were measurable [11]. Jiang et al. focused on synthesizing a mixed robust H_2_/H_∞_ linear parameter-varying controller for the longitudinal motion of an HFV via a high-order singular value decomposition approach [12]. Wang et al. dealt with the second-order dynamic sliding-mode control problem for a non-minimum phase underactuated HFV [2]. Guo et al. established a disturbance estimation-triggered pool scheme for attitude tracking and guaranteed the ultimately bounded stability of the closed-loop system [13]. Most studies on the HFV control systems have only focused on the fixed fuselage without considering the variable- structure aerodynamic areas. Hence, to resolve this deficiency, the current study investigates the control technology of variable-structure HFVs.

During flight, an HFV will inevitably encounter complex electromagnetic or airflow disturbances. Accordingly, the robust control of disturbances is a core aspect of the flight control theory and a critical research subject [14]. Sun et al. proposed a fixed-time convergent non-smooth backstepping control scheme for an HFV via an augmented sliding mode observer to overcome noise [15]. Xu et al. investigated the disturbance observer-based neural adaptive control on the longitudinal HFV dynamics in the presence of wind effects [16]. Ma et al. proposed a fuzzy model predictive controller based on an adaptive neural network disturbance observer for the longitudinal dynamics of a constrained HFV in the presence of diverse disturbances [17]. Hu et al. proposed a robust adaptive fuzzy tracking controller for an HFV subject to both parametric uncertainties and unmodeled dynamics [18]. Some of the foregoing studies relied on robust performance indicators. In other investigations, the estimation algorithm employed was considerably simple to satisfy the flight control target under complex disturbances. In this study, a large-value time-varying disturbance stabilization control is investigated. Moreover, the amplitude limitation caused by the dependence on performance indicators is overcome.

To solve the compensation problems of more complicated faults, fault detection and isolation (FDI), diagnosis, and HFV FTC are mainly implemented. The FDI allows the systems to accurately obtain the time and location of faults [19]. Amato et al. proposed an FDI algorithm to estimate the attitude of an unmanned aerial vehicle (UAV) using low-cost magnetometers and gyroscopes, implemented in an inertial measurement unit [20]. Wang et al. proposed a data-driven multivariate regression approach based on the long short-term memory with residual filtering for the UAV flight data fault detection [21]. Wen et al. presented a novel neural network-based FDI technique applicable to a class of nonlinear systems. The adaptive observer was designed for fault detection based on a single hidden-layer wavelet neural network [22]. The above studies, however, lack the physical background of the HFV. The present study utilizes related methods to investigate the multi-fault FDI technology of HFVs.

Fault diagnosis and FTC enable the aircraft to obtain the fault amplitude and reconfigure the controller as well as achieve self-healing control [23]. Chen et al. developed a novel finite element approach for switched descriptor systems subject to switching actions and state-inconsistence phenomena [24]. Chen et al. studied the fault/bias estimation based on the two-stage Kalman filter and unscented Kalman filter in the presence of unknown random biases [25]. Chen et al. proposed new real-valued timed failure propagation graphs (rTFPGs), designed for continuous-state systems, and presented a systematic method for constructing rTFPGs by combining the capabilities of human experts and data-driven methods [26]. The foregoing studies focused on the fault estimation of general aircrafts without the FTC. This present study considers the particularity of s and formulates an integrated diagnosis variable-structure HFV compensation control scheme. Considerable advances have been achieved in research on HFV fault compensation [27, 28]. Considering the attack angle constraint, Xu et al. investigated the FTC of an HFV using back-stepping and composite learning. The control laws were designed based on the barrier Lyapunov function [29]. In the simultaneous presence of aerodynamic uncertainties, modeling errors, external disturbances, and contingent actuator failures, Yuan et al. proposed an adaptive FTC scheme in the context of dynamic surface control [30]. Meng et al. studied an HFV with the centroid shift and actuator faults to investigate the adaptive FTC for the stability recovery of an HFV operating under off-nominal conditions [31]. However, the studies on the HFV fault compensation seldom consider multi-sensor faults. The present study investigates the multi-sensor fault FTC technology of HFVs. Based on the aforementioned investigations and the related goals of this research, the main contributions of this study are summarized as follows.

- This paper studies the multi-sensor faults of variable structure HFV, which is more general than the study of single fault. As multi-sensor faults is different from the model based on HFV nonlinear dynamics, the type-II fuzzy theory is utilized to simplify the model.
- For the FDI, the output residuals and thresholds are combined to detect and isolate faulty sensors. The design of alarm thresholds considers the observation error, disturbance, and variable-structure parameters.
- An extra differential part is proposed to estimate a fast-varying large amplitude disturbance (FLAD), which avoids the amplitude limitation caused by the use of robust performance indicators. The adaptive parameters that switch with the interference derivative in FTC respond to FLAD in time to compensate for its impact on the fault repair process.
- Both the nominal and fault-tolerant controllers have active variable structure parameters, which enable them to achieve direct adaptive control of the fuselage structure evolution.

The remainder of this paper is arranged as follows. Section 2 presents the dynamics for variable structure HFV with multi-sensor faults and a nominal controller. Section 3 presents the fault diagnosis and FTC schemes. Main results including all stability proofs are given in Section 3. Section 4 conducts simulations to illustrate the validity of the proposed schemes. Section 5 makes a final summary.

## 2. Problem formulation

### 2.1. Variable-structure HFV model

The longitudinal variable-structure HFV model adds variable-structure aerodynamic areas to the classic model. This is described by differential equations with velocity (*V*), flight path angle (*γ*), altitude (*h*), attack angle (*α*), and pitch rate (*q*). The nonlinear model is expressed as [30]:

$$\left\{ \begin{array}{l} \dot{V}=(T\cos\alpha -D_{dr})/m-\rho \sin\gamma /r^{2}\\ \dot{h}=V\sin\gamma \\ \dot{\gamma }=(L+T\sin\alpha )/mV-(\rho -V^{2}r)\cos\gamma /Vr^{2}\\ \dot{\alpha }=q-\dot{\gamma }\\ \dot{q}=M_{yy}/I_{yy} \end{array}\right.$$ (1)

where *T*, *D*_*dr*_, *L*, *m*, *ρ*, *M*_*yy*_, and *I*_*yy*_ denote the thrust, drag, lift, aircraft mass, gravitational constant, pitch moment, and moment of inertia, respectively; *R* represents the earth’s radius*; r* = *R*+*h*. The forces and moments are expressed as

$$\left\{ \begin{array}{l} T=0.5\rho V^{2}(S+S_{s})C_{T}=0.5\rho V^{2}SC_{T}+0.5\rho V^{2}S_{s}C_{T}\\ L=0.5\rho V^{2}(S+S_{s})C_{L}=0.5\rho V^{2}SC_{L}+0.5\rho V^{2}S_{s}C_{L}\\ D_{dr}=0.5\rho V^{2}(S+S_{s})C_{D}=0.5\rho V^{2}SC_{D}+0.5\rho V^{2}S_{s}C_{D}\\ M_{yy}=0.5\rho V^{2}(S+S_{s})C_{\bar{C}}(C_{M}(\alpha )+c_{e}C_{M}(\delta_{e})+0.5c_{q}C_{M}(q)) \end{array}\right.$$ (2)

where *S* is the nominal aerodynamic area; *S*_*s*_ is the variable-structure aerodynamic area; *δ*_*e*_ denotes the elevator deflection control signals; *C*_*T*_, *C*_*D*_, *and C*_*L*_ are the coefficients of throttle, drag, and lift, respectively; *C*_*M*_(**·**) is the pitch moment coefficient with respect to (**·**). More parameter details are presented in [5]; *C*_*T*_ contains another control signal, *δ*_*T*_.


$$C_{T}=\{\begin{array}{l} 0.02576\delta_{T},\;\delta_{T}<1\\ 0.0224+0.00336\;\delta_{T},\delta_{T}\geq 1 \end{array}.$$

In an actual flight, multi-sensor faults may occur when the HFV encounters a harsh environment or its structure changes. However, the HFV longitudinal model (1) has two problems. First, the model is nonlinear and strongly coupled. Multi-sensor fault modeling based on this mode is difficult, and the corresponding high-quality FDI and active FTC are unlikely to be realized. Second, the model parameters are uncertain, as expressed by:

$$\left\{ \begin{array}{l} m=m_{0}(1+\Delta m)\\ I_{yy}=I_{yy0}(1+\Delta I_{yy})\\ S=S_{0}(1+\Delta S)\\ S_{s}=S_{s0}(1+\Delta S_{s})\\ c_{e}=c_{e0}(1+\Delta c_{e})\\ c_{q}=c_{q0}(1+\Delta c_{q})\\ \rho =\rho_{0}(1+\Delta \rho ) \end{array}\right.$$ (3)

where (**·**_0_) are the nominal values, Δ(**·**) are the parameter uncertainties. Solving these two problems requires type-II fuzzy method, which can linearize the nonlinear model, its upper and lower membership functions can eliminate the parameter uncertainties and ensure the model standardization [31]. The controller designed for the simplified model will not need to consider the above two problems.

### 2.2. Fuzzy modeling with multisensor faults

As mentioned earlier, the type-II fuzzy technique is utilized in this study to simplify system (1), which is conducive to the design of diagnosis and compensation schemes.

First, the longitudinal model described in (1) can be written in an affine nonlinear form:

$$\left\{ \begin{array}{l} \dot{x}(t)=f(x)+g(x)u(t)\\ y(t)=Cx(t) \end{array}\right.$$ (4)

where *x*(*t*) = [*x*_1_(*t*), *x*_2_(*t*), *x*_3_(*t*), *x*_4_(*t*), *x*_5_(*t*)]^*T*^ = [*V*, *γ*, *h*, *α*, *q*]^*T*^∈*R*^5^ is the state vector and *u*(*t*) = [*δ*_*e*_, *δ*_*T*_]^*T*^∈*R*^2^ is the control input vector. Given that the HFV has five sensors (i.e., velocity, flight-path angle, altitude, attack angle, and pitch rate sensors) to measure the real-time flight states, the output vector can be derived as *y*(*t*) = [*V*, *γ*, *h*, *α*, *q*]^*T*^∈*R*^5^.

The type-II fuzzy model of variable structure HFV is then expressed as:

*Rule i*: IF *f*_1_(*x*_1_(*t*)) is *ξ*_*i*1_… *f*_5_(*x*_5_(*t*)) is *ξ*_*i*5_, THEN

$$\left\{ \begin{array}{l} \dot{x}(t)=(A_{i}+A_{s})x(t)+(B_{i}+B_{s})u(t)\\ y(t)=C_{i}x(t) \end{array}\right.$$ (5)

where *A*_*i*_, *A*_*s*_, *C*_*i*_∈*R*^5×5^, *B*_*i*_, *B*_*s*_∈*R*^5×2^, *A*_*i*_, *B*_*i*_, *C*_*i*_ are the linear modal parameter matrices, *A*_*s*_, *B*_*s*_ are the active variable structure parameters obtained according to *S*_*s*_ in (1) and (2), *C*_*i*_ represents the nature of output measurement and is not affected by the aerodynamic area structure, *ξ*_*ij*_(*i* = 1,…, *w*; *j* = 1,…, 5) is interval type-II fuzzy set. The triggering strength of each fuzzy rule is expressed as

$$\left\{ \begin{array}{l} \omega_{i}(x(t))=[\omega_{i,low}(x(t)),\omega_{i,up}(x(t))]\\ \omega_{i,up}(x(t))=\prod_{j=1}^{5}\mu_{\xi_{ij},up}(x_{j}(t))\\ \omega_{i,low}(x(t))=\prod_{j=1}^{5}\mu_{\xi_{ij},low}(x_{j}(t)) \end{array}\right.$$ (6)

where *ω*_*i*,*up*_(*x*(*t*))≥*ω*_*i*,*low*_(*x*(*t*))≥0. *ω*_*i*,*up*_(*x*(*t*)) is the upper membership, *ω*_*i*,*low*_(*x*(*t*)) is the lower membership. The upper and lower membership functions are represented by $\mu_{\xi_{ij},up}(x_{j}(t))$ and $\mu_{\xi_{ij},low}(x_{j}(t))$, respectively. The global type-II fuzzy model is expressed as follows:

$$\left\{ \begin{array}{l} \dot{x}(t)=\sum_{i=1}^{w}\left(\varphi_{i,low}+\varphi_{i,up}\right)[(A_{i}+A_{s})x(t)+(B_{i}+B_{s})u(t)]\\ y(t)=\sum_{i=1}^{w}\left(\varphi_{i,low}+\varphi_{i,up}\right)C_{i}x(t) \end{array}\right.$$ (7)

where

$$\begin{array}{l} \varphi_{i,low}=\varphi_{i,low}(x(t))\\ =\frac{\nu_{i}(x(t))\omega_{i,low}(x(t))}{\sum_{i=1}^{w}\left(\nu_{i}(x(t))\omega_{i,low}(x(t))+(1-\nu_{i}(x(t)))\omega_{i,up}(x(t))\right)} \end{array}$$ (8)

$$\begin{array}{l} \varphi_{i,up}=\varphi_{i,up}(x(t))\\ =\frac{\left(1-\nu_{i}(x(t)))\omega_{i,up}(x(t)\right)}{\sum_{i=1}^{w}\left(\nu_{i}(x(t))\omega_{i,low}(x(t))+(1-\nu_{i}(x(t)))\omega_{i,up}(x(t))\right)} \end{array}$$ (9)

and *ν*_*i*_(*x*(*t*)) is the weight coefficient satisfies 0≤*ν*_*i*_(*x*(*t*)) ≤1.

Although the type-II fuzzy technique has simplified the structure and parameters of the nonlinear dynamics of (1), an approximation error between (1) and (5) inevitably exists. Therefore, some nonlinear terms (*g*_*i*_(*x*, *t*)) describing this error are added to improve the model accuracy. In harsh environments, more than one sensor fails at a time. In this study, we only consider two sensors that fail and have bias faults.

In the harsh environment, it is possible that more than one sensor fails at a time. In this study, we only consider two sensors that fail and have bias faults.

Assume that a bias fault in sensor *k* and another bias fault in sensor *s* exist (1≤*k<s*≤5). Moreover, consider the approximation error and FLAD. Then, the variable-structure HFV fuzzy model is described as:

$$\left\{ \begin{array}{l} \dot{x}(t)=\sum_{i=1}^{w}\left(\varphi_{i,low}+\varphi_{i,up}\right)[(A_{i}+A_{s})x(t)+(B_{i}+B_{s})u(t)\\ +g_{i}(x,t)]+Dd(t)\\ y^{f}(t)=\sum_{i=1}^{w}\left(\varphi_{i,low}+\varphi_{i,up}\right)C_{i}x(t)+Ff(t)+F_{\omega }\omega (t) \end{array}\right.$$ (10)

where *g*_*i*_(*x*, *t*) is a smooth vector field on *R*^5^; *d*(*t*)∈*R*^1^, *f*(*t*)∈*R*^2^, and *ω*(*t*)∈*R*^1^ denote the input FLAD, sensor faults, and output noise, respectively; *F*∈*R*^5×2^ (*F*_*k*1_ = 1, *F*_*s*2_ = 1, and other entries of *F* are equal to zero) is an unknown matrix; *D*∈*R*^5^ and *F*_*ω*_∈*R*^5^ are two constant matrices.

*Assumption 1*. Pairs (*A*_*i*_, *A*_*s*_, *B*_*i*_, *B*_*s*_) is completely controllable. Pairs (*A*_*i*_, *A*_*s*_, *C*_*i*_) is observable.

*Assumption 2*. ||*d*(*t*)||≤*d*_0_, $\|\dot{d}(t)\|\leq d_{1},\;\|g_{i}(x,\;t)\|\leq g_{0}\;\mathrm{and}\;\|{\dot{g}}_{i}(x,t)\|\leq g_{1}$, *d*_0_, *d*_1_, *g*_0_, and *g*_1_ are positive scalars.

*Assumption 3*. The nonlinear term *g*_*i*_(*x*, *t*) can satisfy:

$$\|g_{i}(x_{1},t)-g_{i}(x_{2},t)\|\leq \mu_{i}\|x_{1}-x_{2}\|,\forall x_{1},x_{2},t$$ (11)

where *μ*_*i*_ is the Lipschitz constant.

*Remark 1*. To enable the designed algorithm to affect the system, we assume that the system can be controlled by the input; to be able to use detection errors to design algorithm and observers, we assume that the system can be observed. The disturbance and nonlinear errors need to be assumed to be bounded, and their respective derivatives are also bounded. The boundedness assumption is easy to understand. The amplitude of any disturbance cannot be infinite, and a large enough amplitude will directly destroy the system. It is unrealistic to study such disturbance. It is necessary to assume that the nonlinear function satisfies the Lipschitz condition, that is, the inequality (11), adjust *μ*_*i*_ so that the right side of the inequality is less than zero. Non-linear functions that do not satisfy Lipschitz condition are usually discontinuous, we do not consider the discrete systems.

Lemma 1 [28]. For any positive scalar *μ* and real matrices *X*, *Y* with appropriate dimensions, (12) holds:

$$X^{T}Y+Y^{T}X\leq \mu X^{T}X+\mu^{-1}Y^{T}Y$$ (12)

### 2.3. Nominal controller design

A controller is designed to ensure system stability and track the commands of altitude and velocity under variable fuselage conditions.

Under the nominal condition without faults and disturbance, let *e*_*yd*_ *= y−y*_*d*_, where *y*_*d*_ is the desired output, and a step signal. An global fuzzy variable-structure controller is selected:

$$\begin{array}{l} u=-\overset{w}{\underset{i=1}{\sum }}(\varphi_{i,\mathrm{low}}+\varphi_{i,up})\{K_{i}e_{yd}+{\left(B_{i}+B_{s}\right)}^{+}[v_{i}\\ +(A_{i}+A_{s})y_{d}]\} \end{array}$$ (13)

$${\dot{v}}_{i}=\Gamma_{1}P_{c}(e_{yd}+{\dot{e}}_{yd})$$ (14)

where *K*_*i*_∈*R*^2×5^ is the output feedback matrix to be designed and *B*_*i*_^*+*^ is the pseudo-inverse of *B*_*i*_. Then, Theorem 1 is obtained.

Theorem 1. *If symmetric positive definite matrices P*_*c*_, *G*, *Γ*_*1*_ *and real matrix K*_*i*_ *exist with appropriate dimensions*, *such that the following inequality holds for any i =* 1, *…*, *w*:

$$[\begin{array}{cc} \Theta_{1} & \Theta_{2}\\ {}* & G-2P_{c} \end{array}]<0$$ (15)

$$\begin{array}{l} \Theta_{1}={\left[(A_{i}+A_{s})-(B_{i}+B_{s})K_{i}\right]}^{T}P_{c}\\ +P_{c}[(A_{i}+A_{s})-(B_{i}+B_{s})K_{i}] \end{array}$$ (16)

$$\Theta_{2}=-{\left[(A_{i}+A_{s})-(B_{i}+B_{s})K_{i}\right]}^{T}P_{c}$$ (17)

then, the tracking error e_yd_ is uniformly ultimately bounded under the controller (13).

*Proof*: Denote

$$e_{gi}=g_{i}(x,t)-v_{i}$$ (18)

The Lyapunov function is selected as follows:

$$V_{c}=e_{yd}^{T}P_{c}e_{yd}+\sum_{i=1}^{w}\left(\varphi_{i,low}+\varphi_{i,up}\right)(e_{gi}^{T}\Gamma_{1}^{-1}e_{gi})$$ (19)

The following error dynamics can be deduced from (10), (13), and (18):

$${\dot{e}}_{yd}=\sum_{i=1}^{w}\left(\varphi_{i,low}+\varphi_{i,up}\right)\{[(A_{i}+A_{s})-(B_{i}+B_{s})K_{i}]e_{yd}+e_{gi}\}$$ (20)

The derivative of (19) can be written as follows:

$$\begin{array}{l} {\dot{V}}_{c}=\sum_{i=1}^{w}\left(\varphi_{i,low}+\varphi_{i,up}\right)\{e_{yd}^{T}[{\left((A_{i}+A_{s})-(B_{i}+B_{s})K_{i}\right)}^{T}P_{c}\\ +P_{c}((A_{i}+A_{s})-(B_{i}+B_{s})K_{i})]e_{yd}+2e_{yd}^{T}P_{c}e_{gi}\\ +2e_{gi}^{T}\Gamma_{1}^{-1}({\dot{g}}_{i}(x,t)-{\dot{v}}_{i})\}\\ =\sum_{i=1}^{w}\left(\varphi_{i,low}+\varphi_{i,up}\right)\{e_{yd}^{T}[{\left((A_{i}+A_{s})-(B_{i}+B_{s})K_{i}\right)}^{T}P_{c}\\ +P_{c}((A_{i}+A_{s})-(B_{i}+B_{s})K_{i})]e_{yd}-2e_{gi}^{T}P_{c}[\left(A_{i}+A_{s}\right)\\ -(B_{i}+B_{s})K_{i}]e_{yd}-2e_{gi}^{T}P_{c}e_{gi}+2e_{g_{i}}^{T}\Gamma_{1}^{-1}{\dot{g}}_{i}(x,t)\} \end{array}$$ (21)

Based on Lemma 1, there exists a matrix *G* = *G*^*T*^>0 such that

$$\begin{array}{l} 2e_{gi}^{T}\Gamma_{1}^{-1}{\dot{g}}_{i}(x,t)\leq e_{gi}^{T}Ge_{gi}+{\dot{g}}_{i}^{T}(x,t)\Gamma_{1}^{-1}G^{-1}\Gamma_{1}^{-1}{\dot{g}}_{i}(x,t)\\ \leq e_{gi}^{T}Ge_{gi}+g_{1}^{2}\max(eig(\Gamma_{1}^{-1}G^{-1}\Gamma_{1}^{-1})) \end{array}$$ (22)

Let

$${\bar{e}}_{ci}={\left[\begin{array}{cc} e_{yd}^{T} & e_{gi}^{T} \end{array}\right]}^{T}$$ (23)

$$\eta_{1}=g_{1}^{2}\max(eig(\Gamma_{1}^{-1}G^{-1}\Gamma_{1}^{-1}))$$ (24)

We have

$$\begin{array}{l} {\dot{V}}_{c}\leq \sum_{i=1}^{w}\left(\varphi_{i,low}+\varphi_{i,up}\right)\\ \{{\bar{e}}_{ci}^{T}[\begin{array}{c} {\left[(A_{i}+A_{s})-(B_{i}+B_{s})K_{i}\right]}^{T}P_{c}+P_{c}[(A_{i}+A_{s})-(B_{i}+B_{s})K_{i}]\\ {}* \end{array}\\ \left[\begin{array}{c} -{\left[(A_{i}+A_{s})-(B_{i}+B_{s})K_{i}\right]}^{T}P_{c}\\ G-2P_{c} \end{array}]{\bar{e}}_{ci}+\eta_{1}\right\} \end{array}$$ (25)

Let

$$\begin{array}{l} \Pi_{i}=[\begin{array}{c} {\left[(A_{i}+A_{s})-(B_{i}+B_{s})K_{i}\right]}^{T}P_{c}+P_{c}[(A_{i}+A_{s})-(B_{i}+B_{s})K_{i}]\\ {}* \end{array}\\ {}[\begin{array}{c} -{\left[(A_{i}+A_{s})-(B_{i}+B_{s})K_{i}\right]}^{T}P_{c}\\ G-2P_{c} \end{array}] \end{array}$$ (26)

Then, (25) can be rewritten as follows:

$${\dot{V}}_{c}\leq \sum_{i=1}^{w}\left(\varphi_{i,low}+\varphi_{i,up}\right)({\bar{e}}_{ci}^{T}\Pi_{i}e_{ci}+\eta_{1})$$ (27)

If (15) holds, then denote *є*_*i*_ = min(eig(−Π_*i*_)). Thus,

$${\dot{V}}_{c}\leq \sum_{i=1}^{w}\left(\varphi_{i,low}+\varphi_{i,up}\right)(-\epsilon_{i}{\left\|{\bar{e}}_{ci}\right\|}^{2}+\eta_{1})$$ (28)

For *i* = 1,…, *w*, when $\|{\bar{e}}_{ci}{\|}^{2}>\eta_{1}/\epsilon_{i}$, ${\dot{V}}_{c}<0$ can be obtained, i.e., the error dynamics converge to an interval:

$$\left\{{\bar{e}}_{ci}(t){\left\|{\bar{e}}_{ci}\right\|}^{2}\leq \eta_{1}/\epsilon_{i}\right\}$$ (29)

Therefore, *e*_*yd*_ and *e*_*gi*_ are ultimately uniformly bounded. This completes the proof of Theorem 1.

The subsequent section presents the design of the diagnosis and FTC schemes to ensure the stability and tracking accuracy of the variable-structure HFV with multi-sensor faults.

## 3. Fault diagnosis and compensation

**Fig 1:**
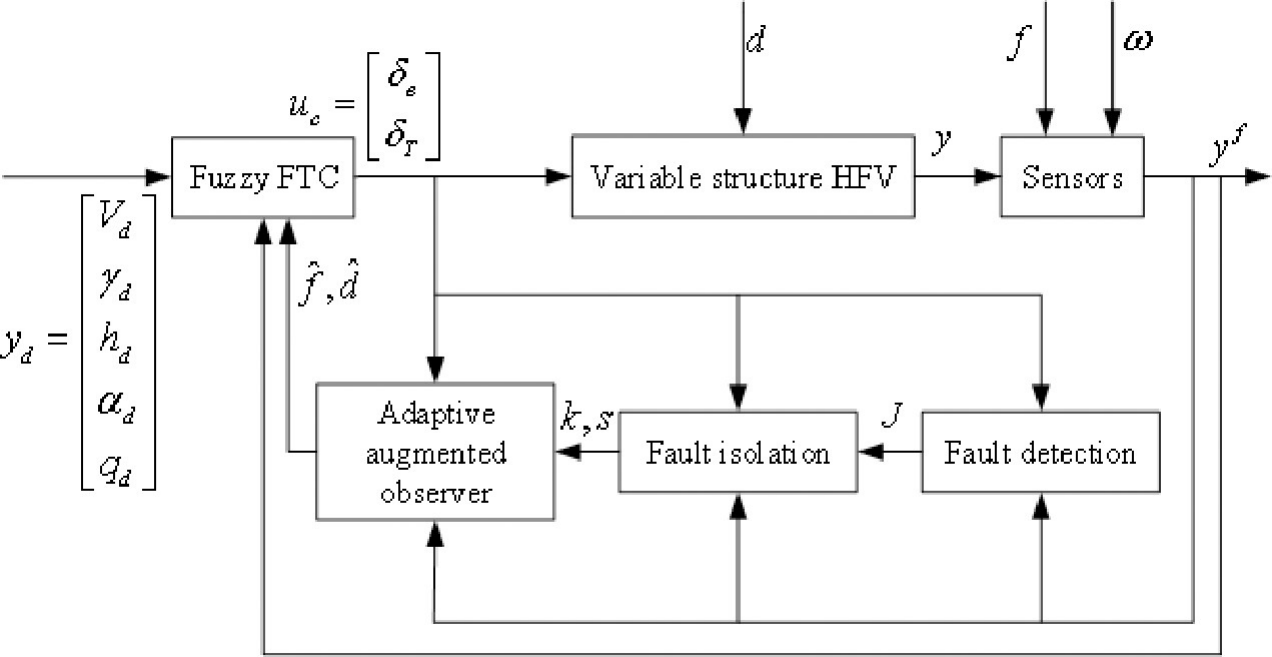
Overall control structure diagram of HFV.

An FTC with the combined nominal controller and fault diagnosis results is developed. An observer-based scheme is implemented to generate a set of sensor output residuals and design thresholds for the FDI. Then, an adaptive augmented observer is formulated to estimate the faults. Through a proportional-differential part, an FLAD whose magnitude may be large under Assumption 2 is also handled. Finally, a robust output feedback FTC is employed to compensate for the faults. Fig 1 shows the entire control structure.

### 3.1. Fault detection

To simplify the design of the variable-structure detection observer, the input FLAD and output noise are not considered. The observer is introduced as follows:

$$\left\{ \begin{array}{l} \dot{\hat{x}}(t)=\sum_{i=1}^{w}\left(\varphi_{i,low}+\varphi_{i,up}\right)[(A_{i}+A_{s})\hat{x}(t)+(B_{i}+B_{s})u(t)\\ +g_{i}(\hat{x},t)+H_{i}(\hat{y}(t)-y^{f}(t))]\\ \hat{y}(t)=\sum_{i=1}^{w}\left(\varphi_{i,low}+\varphi_{i,up}\right)C_{i}\hat{x}(t) \end{array}\right.$$ (30)

where *H*_*i*_∈*R*^5×5^ is the observer gain matrix to be designed.

The actual corresponding functions are subtracted from the observed state/output functions. The errors are denoted as follows:

$$\left\{ \begin{array}{l} e_{x}(t)=\hat{x}(t)-x(t)\\ e_{y}(t)=\hat{y}(t)-y^{f}(t) \end{array}\right.$$ (31)

Theorem 2. *Under the fault-free condition*, *(y*^*f*^(*t*) *= y*(*t*)*)*, *if there exists a positive scalar κ*_*d*_, *symmetric positive definite matrices P*_*d*_, *and Q*_*d*_, *and a real matrix Y*_*i*_ *with appropriate dimensions*, *such that the following inequality holds with H*_*i*_ *= P*_*d*_^*−1*^*Y*_*i*_, *then the estimate error*, *e*_*x*_(*t*), *asymptotically converges to zero*.


$$\begin{array}{l} \sum_{i=1}^{w}\left(\varphi_{i,low}+\varphi_{i,up}\right)\\ \left[\begin{array}{cc} P_{d}(A_{i}+A_{s})+{\left(A_{i}+A_{s}\right)}^{T}P_{d}+Y_{i}C+C^{T}Y_{i}^{T}+\kappa_{d}\mu_{i}^{2}I & P_{d}\\ {}* & -\kappa_{d}I \end{array}\right]\\ <-Q_{d} \end{array}$$ (32)

*Proof*: The Lyapunov function is selected as follows:

$$V_{d}=e_{x}^{T}P_{d}e_{x}$$ (33)

$$\begin{array}{l} {\dot{e}}_{x}=\dot{\hat{x}}-\dot{x}\\ =\sum_{i=1}^{w}\left(\varphi_{i,low}+\varphi_{i,up}\right)[(A_{i}+A_{s}+H_{i}C_{i})e_{x}\\ +g_{i}(\hat{x},t)-g_{i}(x,t)] \end{array}$$ (34)

Referring to (34), the derivative of (33) can be written as follows:

$$\begin{array}{l} {\dot{V}}_{d}=\sum_{i=1}^{w}\left(\varphi_{i,low}+\varphi_{i,up}\right)\{e_{x}^{T}[P_{d}(A_{i}+A_{s}+H_{i}C_{i})+(A_{i}\\ +A_{s}+H_{i}C_{i}{)}^{T}P_{d}]e_{x}+2e_{x}^{T}P_{d}(g_{i}(\hat{x},t)-g_{i}(x,t))\}\\ =\sum_{i=1}^{w}\left(\varphi_{i,low}+\varphi_{i,up}\right)\{e_{x}^{T}[P_{d}(A_{i}+A_{s})+{\left(A_{i}+A_{s}\right)}^{T}P_{d}\\ +Y_{i}C_{i}+C_{i}^{T}Y_{i}^{T}]e_{x}+2e_{x}^{T}P_{d}(g_{i}(\hat{x},t)-g_{i}(x,t))\}\\ \leq \sum_{i=1}^{w}\left(\varphi_{i,low}+\varphi_{i,up}\right)\{e_{x}^{T}[P_{d}(A_{i}+A_{s})+{\left(A_{i}+A_{s}\right)}^{T}P_{d}\\ +Y_{i}C_{i}+C_{i}^{T}Y_{i}^{T}]e_{x}+\kappa_{d}^{-1}e_{x}^{T}P_{d}^{2}e_{x}+\kappa_{d}{\left\|g_{i}(\hat{x},t)-g_{i}(x,t)\right\|}^{2}\}\\ \leq \sum_{i=1}^{w}\left(\varphi_{i,low}+\varphi_{i,up}\right)\{e_{x}^{T}[P_{d}(A_{i}+A_{s})+{\left(A_{i}+A_{s}\right)}^{T}P_{d}\\ +Y_{i}C_{i}+C_{i}^{T}Y_{i}^{T}+\kappa_{d}^{-1}P_{d}^{2}+\kappa_{d}\mu_{i}^{2}I]e_{x}\} \end{array}$$ (35)

Consider the following inequality:

$$\begin{array}{l} \overset{w}{\underset{i=1}{\sum }}(\varphi_{i,low}+\varphi_{i,up})(P_{d}(A_{i}+A_{s})+{\left(A_{i}+A_{s}\right)}^{T}P_{d}\\ +Y_{i}C_{i}+C_{i}^{T}Y_{i}^{T}+\kappa_{d}^{-1}P_{d}^{2}+\kappa_{d}\mu_{i}^{2}I)<-Q_{d} \end{array}$$ (36)

If the inequality holds, then, ${\dot{V}}_{d}<0$ can be obtained. The detection system is stable.

According to the Schur complement, if (32) holds, then lim_*t*→∞_*e*_*x*_(*t*) = 0.

The detection residual and detection mechanism are defined as follows:

$$r(t)=\|\hat{y}(t)-y^{f}(t)\|=\|e_{y}(t)\|$$ (37)

$$r(t)\{\begin{array}{l} \leq J,no\;faults\;occurred\\ >J,faults\;have\;occurred \end{array}$$ (38)

The value of threshold *J* is important for fault detection because it is associated with missing and false reports. Next, *J* is designed by considering the observer error (30) and external FLAD.

From Lemma 2, we obtain

$$\begin{array}{l} {\dot{V}}_{d}<-\sum_{i=1}^{w}\left(\varphi_{i,low}+\varphi_{i,up}\right)(e_{x}^{T}Q_{d}e_{x})\\ \leq -\frac{\min(\mathrm{eig}(Q_{d}))}{\max(\mathrm{eig}(P_{d}))}\sum_{i=1}^{w}\left(\varphi_{i,low}+\varphi_{i,up}\right)(e_{x}^{T}P_{d}e_{x})\\ \leq -\frac{\min(\mathrm{eig}(Q_{d}))}{\max(\mathrm{eig}(P_{d}))}V_{d} \end{array}$$ (39)

Let

$$\frac{\min(\mathrm{eig}(Q_{d}))}{\max(\mathrm{eig}(P_{d}))}=\lambda (\lambda >0)$$ (40)

Then

$$\left\{ \begin{array}{l} {\dot{V}}_{d}(t)\leq -\lambda V_{d}(t)\\ V_{d}(t)\leq e^{-\lambda t}V_{d}(0) \end{array}\right.$$ (41)

By combining (33) and (41), we can derive (42):

$$\min(\mathrm{eig}(P_{d})){\left\|e_{x}(t)\right\|}^{2}\leq e^{-\lambda t}\max(\mathrm{eig}(P_{d})){\left\|e_{x}(0)\right\|}^{2}$$ (42)

Then,

$$\|e_{x}(t)\|\leq \sqrt{\max(eig(P_{d}))/\min(eig(P_{d}))}\|e_{x}(0)\|e^{-\lambda t/2}$$ (43)

Note that *r*(0) can be used to substitute ||*C*_*i*_||||*e*_*x*_(0)||. Under the fault and disturbance-free circumstances, that is, *y*^*f*^(*t*) *= y*(*t*), *d*(*t*) = 0, and *ω*(*t*) = 0,

$$\begin{array}{l} r(t)=\|\hat{y}(t)-y^{f}(t)\|\\ =\sum_{i=1}^{w}\left(\varphi_{i,low}+\varphi_{i,up}\right)\|C_{i}e_{x}(t)\|\\ \leq \sum_{i=1}^{w}\left(\varphi_{i,low}+\varphi_{i,up}\right)\|C_{i}\|\sqrt{\max(eig(P_{d}))/min(eig(P_{d}))}\|e_{x}(0)\|e^{-\lambda t/2}\\ \leq \sqrt{\max(eig(P_{d}))/min(eig(P_{d}))}\|r(0)\|e^{-\lambda t/2} \end{array}$$ (44)

Denote

$$J_{0}=\sqrt{\max(eig(P_{d}))/min(eig(P_{d}))}\|r(0)\|e^{-\lambda t/2}$$ (45)

When considering *d*(*t*) and *ω*(*t*), the detection threshold is derived as follows:

$$J=\beta J_{0}+\varepsilon$$ (46)

where *β*>1 and *ε*>0 are two constants whose values are determined by the magnitudes of FLAD and noise.

The detection alarm activates when the sensor output residual exceeds the threshold, which is set in advance.

### 3.2. Fault isolation

Although it can be determined whether sensor faults exist in the variable-structure HFV over time, it cannot be determined which sensors have failed only through detection. Therefore, fault isolation is necessary.

Existing methods are difficult to isolate multiple faults. Accordingly, in this section, an improved isolation process is proposed. In accordance with the concept of combination, more observers are necessary to isolate multi-sensor faults from a single fault. As mentioned, it is necessary to design 10 isolation observers because two sensors are assumed to fail. Next, the improved isolation process of multi-sensor fault isolation is explained in detail.

First, the following is defined:

$$\left\{ \begin{array}{l} y_{ks}^{r}≜y\backslash (y_{k}\&y_{s})\\ C_{ks,i}^{r}≜C_{i}\backslash (C_{k,i}\&C_{s,i})\\ H_{ks,i}^{r}≜H_{i}\backslash (H_{k,i}\&H_{s,i}) \end{array}\right.$$ (47)

where *y*^*r*^_*ks*_, *C*^*r*^_*ks*,*i*_, and *H*^*r*^_*ks*,*i*_ are the remaining parts of *y*, *C*_*i*_, and *H*_*i*_, respectively, after the deletion of their *k*th and *s*th rows.

For 1≤*k*<*s*≤5, the *m*th (*m* = 1, …, 10) variable- structure isolation observer is introduced as follows:

$$\left\{ \begin{array}{l} {\dot{\hat{x}}}_{m}(t)=\sum_{i=1}^{w}\left(\varphi_{i,low}+\varphi_{i,up}\right)[(A_{i}+A_{s}){\hat{x}}_{m}(t)+(B_{i}\\ +B_{s})u(t)+g_{i}(\hat{x},t)+H_{ks,i}^{r}({\hat{y}}_{ks}^{r}-y_{ks}^{r,f})]\\ {\hat{y}}_{m}(t)=\sum_{i=1}^{w}\left(\varphi_{i,low}+\varphi_{i,up}\right)C_{i}{\hat{x}}_{m}(t) \end{array}\right.$$ (48)

Define

$$\left\{ \begin{array}{l} r_{m}(t)=\|{\hat{y}}_{m}-y_{m}^{f}\|\\ r_{m}^{r}(t)=\|{\hat{y}}_{ks}^{r}-y_{ks}^{r,f}\| \end{array}\right.$$ (49)

$$e_{xm}(t)={\hat{x}}_{m}(t)-x_{m}(t)$$ (50)

Based on Theorem 2, if inequality (44) holds such that *H*^*r*^_*ks*,*i*_ = *P*_*m*_^−1^ *C*^*r*^_*ks*,*i*_ for any *i* = 1, …, *w*,

$$\sum_{i=1}^{w}\left(\varphi_{i,low}+\varphi_{i,up}\right)[\begin{array}{cc} \Theta_{3} & P_{m}\\ {}* & -\kappa_{m}I \end{array}]<-Q_{m}$$ (51)

$$\begin{array}{l} \Theta_{3}=P_{m}(A_{i}+A_{s})+{\left(A_{i}+A_{s}\right)}^{T}P_{m}+Y_{ks,i}^{r}C_{ks,i}^{r}\\ +{\left(C_{ks,i}^{r}\right)}^{T}{\left(Y_{ks,i}^{r}\right)}^{T}+\kappa_{m}\mu_{i}^{2}I \end{array}$$ (52)

then the estimate error *e*_*xm*_(*t*) asymptotically converges to zero.

Similarly, we define

$$\left\{ \begin{array}{l} J_{0m}=\sqrt{\max(eig(P_{m}))/min(eig(P_{m}))}\|r_{m}(0)\|e^{-\lambda_{m}t/2}\\ J_{0m}^{r}=\sqrt{\max(eig(P_{m}))/min(eig(P_{m}))}\|r_{m}^{r}(0)\|e^{-\lambda_{m}t/2} \end{array}\right.$$ (53)

$$\left\{ \begin{array}{l} J_{m}=\beta_{m}J_{0m}+\varepsilon_{m}\\ J_{m}^{r}=\beta_{m}J_{0m}^{r}+\varepsilon_{m} \end{array}\right.$$ (54)

where

$$\left\{ \begin{array}{l} \lambda_{m}=\frac{\min(eig(Q_{m}))}{\max(eig(P_{m}))}\\ r_{m}^{r}(0)\approx \sum_{i=1}^{w}\left(\varphi_{i,low}+\varphi_{i,up}\right)\|C_{ks,i}^{r}\|\|e_{xm}(0)\| \end{array}\right.$$ (55)

Thus, the fault isolation mechanism is presented as:

$$\left\{ \begin{array}{l} r_{m}^{r}(t)\leq J_{m}^{r}\\ r_{m}(t)>J_{m} \end{array}\right.$$ (56)

Hence, the *m*th observer is capable of isolating the faulty sensors, i.e., the *k*th and *s*th sensors are known to have failed, and the unknown matrix *F* is determined.

This study only considers the situation where two sensor faults occur instead of one or more than two sensor failures; hence, the 10 sets of thresholds are independent of each other. When faults occur in sensors *k* and *s*, only the observer with the exact matrices, *y*^*r*^_*ks*_*C*^*r*^_*ks*,*i*_ and *H*^*r*^_*ks*,*i*_, can isolate the faulty sensors because the residual (*r*_*m*_^*r*^) generated by this observer is always less than *J*_*m*_^*r*^. The other residuals exceed their corresponding thresholds after the sensor faults occur.

Hence, faulty sensors are identified, and an effective estimation scheme should be designed to obtain their fault values rapidly and accurately.

### 3.3. Fault estimation

This section presents an adaptive augmented observer developed to accurately estimate the magnitude of these two sensor faults when FLAD exist in preparation for the subsequent fault compensation. To make the magnitude of disturbance unrestricted under the premise that the FLAD is bounded, the type-II fuzzy adaptive estimation method is employed instead of using the performance indicators. Notably, an adaptive law with an improved proportional-differential part estimates a class of FLAD; its performance is compared with the method in [26].

The augmented method is also used for the estimation. Parallel fault estimation considers the combinatorial effects of faulty sensors; this is more reasonable. The variable-structure HFV model with sensor faults, FLAD, and output noise can be rewritten in the following augmented form:

$$\left\{ \begin{array}{l} E{\dot{x}}^{r}(t)=\sum_{i=1}^{w}\left(\varphi_{i,low}+\varphi_{i,up}\right)[(A_{au,i}+A_{au,s})x_{au}(t)+(B_{i}\\ +B_{s})u(t)+g_{i}(\hat{x},t)]+Dd(t)\\ y^{f}=\sum_{i=1}^{w}\left(\varphi_{i,low}+\varphi_{i,up}\right)C_{au,i}x_{au}(t) \end{array}\right.$$ (57)

where

$$\left\{ \begin{array}{l} x_{au}(t)={\left[x^{T}(t)\;x_{f\omega }^{T}(t)\right]}^{T},x_{f\omega }(t)=Ff(t)+F_{\omega }\omega (t)\\ A_{au,i}=[A_{i}\;0_{5\times 5}],A_{au,s}=[A_{s}\;0_{5\times 5}]\\ C_{au,i}=[C_{i}\;I_{5}]\\ F_{au}=[F\;F_{\omega }]\\ E=[I_{5}\;0_{5\times 5}] \end{array}\right.$$ (58)

Let

$$\begin{array}{l} W=\sum_{i=1}^{w}\left(\varphi_{i,low}+\varphi_{i,up}\right)\left[\begin{array}{c} E\\ C_{au,i} \end{array}\right]\\ =\sum_{i=1}^{w}\left(\varphi_{i,low}+\varphi_{i,up}\right)\left[\begin{array}{cc} I_{5} & 0_{5\times 5}\\ C_{i} & I_{5} \end{array}\right] \end{array}$$ (59)

$$\{\begin{array}{l} U={\left(W^{T}W\right)}^{-1}{\left[I_{5}^{T}0_{5\times 5}\right]}^{T}\\ V={\left(W^{T}W\right)}^{-1}\overset{w}{\underset{i=1}{\sum }}(\varphi_{i,low}+\varphi_{i,up}){\left[C_{i}^{T}\;I_{5}^{T}\right]}^{T} \end{array}$$ (60)

To estimate multisensor faults *f*(*t*) and the FLAD *d*(*t*), the following adaptive augmented observer is designed:

$$\left\{ \begin{array}{l} \dot{\xi }=\sum_{i=1}^{w}\left(\varphi_{i,low}+\varphi_{i,up}\right)[(M_{i}+M_{s})\xi +(N_{i}+N_{s})y^{f}\\ +U^{*}(B_{i}+B_{s})u+U^{*}g_{i}(\hat{x},t)+U^{*}D\hat{d}]\\ {\hat{x}}_{au}=\xi +Vy^{f}\\ \hat{y}=\sum_{i=1}^{w}\left(\varphi_{i,low}+\varphi_{i,up}\right)C_{au,i}{\hat{x}}_{au} \end{array}\right.$$ (61)

where *ξ* is a fictitious variable with respect to the augmented vector ^x^au; *M*_*i*_, *N*_*i*_ and *M*_*s*_, *N*_*s*_ are the fixed structure gain matrices and variable structure gain matrices to be determined later, respectively. U^*^ satisfy:

$$U^{*}=\Theta U=\vartheta_{1}\dot{\hat{d}}\vartheta_{2}U$$ (62)

where Θ is the fast adaptive compensation function, *ϑ*_1_ and *ϑ*_2_ are the learning rates. Adding the derivative of FLAD estimation in Θ can make the observer more sensitive to the rate of disturbance change, thereby adapting to the speed of FLAD and compensating it, eliminating FLAD’s influence on fault estimation.

The following error vectors are defined:

$$\left\{ \begin{array}{l} e_{au}(t)={\hat{x}}_{au}(t)-x_{au}(t)\\ e_{d}(t)=\hat{d}(t)-d(t) \end{array}\right.$$ (63)

By considering (60)–(63), we can obtain

$$\begin{array}{l} {\dot{e}}_{au}=\dot{\xi }+V\sum_{i=1}^{w}\left(\varphi_{i,low}+\varphi_{i,up}\right)C_{au,i}{\dot{x}}_{au}-{\dot{x}}_{au}=\dot{\xi }-U^{*}E{\dot{x}}_{au}\\ =\sum_{i=1}^{w}\left(\varphi_{i,low}+\varphi_{i,up}\right)[(M_{i}+M_{s})\xi +(N_{i}+N_{s})y^{f}\\ +\Theta U(B_{i}+B_{s})u+\Theta Ug_{i}(\hat{x},t)-\Theta U(A_{au,i}+A_{au,s})x_{au}\\ -\Theta U(B_{i}+B_{s})u-\Theta Ug_{i}(x,t)]+UDe_{d}\\ =\sum_{i=1}^{w}\left(\varphi_{i,low}+\varphi_{i,up}\right)[(M_{i}+M_{s})({\hat{x}}_{au}-VC_{au,i}x_{au})\\ +(N_{i}+N_{s})C_{au,i}x_{au}-\vartheta_{1}\dot{\hat{d}}\vartheta_{2}U(A_{au,i}+A_{au,s})x_{au}\\ +\vartheta_{1}\dot{\hat{d}}\vartheta_{2}U(g_{i}(\hat{x},t)-g_{i}(x,t))]+\vartheta_{1}\dot{\hat{d}}\vartheta_{2}UDe_{d}\\ =\sum_{i=1}^{w}\left(\varphi_{i,low}+\varphi_{i,up}\right)\{(M_{i}+M_{s})e_{au}+[M_{i}+M_{s}\\ -(M_{i}+M_{s})VC_{au,i}+(N_{i}+N_{s})C_{au,i}-\vartheta_{1}\dot{\hat{d}}\vartheta_{2}U(A_{au,i}\\ +A_{au,s})]x_{au}+\vartheta_{1}\dot{\hat{d}}\vartheta_{2}U(g_{i}(\hat{x},t)-g_{i}(x,t))\}+\vartheta_{1}\dot{\hat{d}}\vartheta_{2}UDe_{d} \end{array}$$ (64)

Let

$$\left\{ \begin{array}{l} N_{i}+N_{s}=R_{i}+(M_{i}+M_{s})V\\ =R_{i}+M_{i}V+M_{s}V\\ M_{i}+M_{s}=\vartheta_{1}\dot{\hat{d}}\vartheta_{2}U(A_{au,i}+A_{au,s})-R_{i}C_{au,i}\\ =\vartheta_{1}\dot{\hat{d}}\vartheta_{2}UA_{au,i}-R_{i}C_{au,i}+\vartheta_{1}\dot{\hat{d}}\vartheta_{2}UA_{au,s} \end{array}\right.$$ (65)

and

$$\left\{ \begin{array}{l} M_{i}=\vartheta_{1}\dot{\hat{d}}\vartheta_{2}UA_{au,i}-R_{i}C_{au,i}\\ M_{s}=\vartheta_{1}\dot{\hat{d}}\vartheta_{2}UA_{au,s}\\ N_{i}=R_{i}+M_{i}V\\ N_{s}=M_{s}V \end{array}\right.$$ (66)

Then

$$\begin{array}{l} {\dot{e}}_{au}=\sum_{i=1}^{w}\left(\varphi_{i,low}+\varphi_{i,up}\right)[(M_{i}+M_{s})e_{au}+\vartheta_{1}\dot{\hat{d}}\vartheta_{2}U(g_{i}(\hat{x},t)\\ -g_{i}(x,t))+\vartheta_{1}\dot{\hat{d}}\vartheta_{2}UDe_{d}] \end{array}$$ (67)

To handle disturbance, robust performance indicators are usually adopted. If there is a high demand for estimation accuracy, the performance indicator must be small when the magnitude of the disturbance is large (For example FLAD). In this case, the LMI and indicator usually have no solution. Furthermore, obtaining the disturbance value aids in the FTC design. Accordingly, an improved adaptive law is proposed to estimate the FLAD as accurately as possible.

The FLAD estimation algorithm is shown as follows:

$$\dot{\hat{d}}=-\Gamma_{2}(e_{y}+{\dot{e}}_{y})$$ (68)

According to definition (31), *e*_*y*_ can be obtained by subtracting the augmented observer output $\hat{y}(t)$ and the sensor output *y*^*f*^(*t*). ${\dot{e}}_{y}$ can be obtained by differentiating *e*_*y*_ with respect to *t*. Given that *e*_*y*_ and ${\dot{e}}_{y}$ are both available, the estimation of FLAD (68) is possible and practical.

In accordance with (57), (58), and (61), (68) can be rewritten as follows:

$$\dot{\hat{d}}=\sum_{i=1}^{w}\left(\varphi_{i,low}+\varphi_{i,up}\right)[-\Gamma_{2}C_{au,i}(e_{au}+{\dot{e}}_{au})]$$ (69)

*Remark 2*. FLAD are estimated using (68) with a differential part, whereas only constant disturbances can be estimated by the proportional-integral (PI) observer in [26]. The adaptive augmented observer discussed in this study can simultaneously estimate the sensor and actuator faults if the FLAD is treated as an actuator fault. The selection basis of Γ_2_ is (70), because (69) is associated with the estimated value of FLAD and Γ_2_, (70) contains this value.

Theorem 3. *If there exists a positive scalar κ*_*e*_, *symmetric positive-definite matrices P*_*1*_, *P*_*2*_, *S*_*1*_, *S*_*2*_, *Γ*_*2*_, *and real matrix M*_*i*_ *with appropriate dimensions*, *such that (70) always holds*:

$$[\begin{array}{ccc} \Psi_{i1} & \Psi_{i2} & P_{1}\vartheta_{1}\dot{\hat{d}}\vartheta_{2}U\\ {}* & \Psi_{i3} & -P_{2}C_{au,i}\vartheta_{1}\dot{\hat{d}}\vartheta_{2}U\\ {}* & * & -\kappa_{e}I \end{array}]<0$$ (70)

$$\Psi_{i1}={\left(M_{i}+M_{s}\right)}^{T}P_{1}+P_{1}(M_{i}+M_{s})+S_{1}+\kappa_{e}\mu_{i}^{2}I$$ (71)

$$\Psi_{i2}=P_{1}\vartheta_{1}\dot{\hat{d}}\vartheta_{2}UD-{\left(M_{i}+M_{s}\right)}^{T}C_{au,i}^{T}P_{2}-C_{au,i}^{T}P_{2}$$ (72)

$$\Psi_{i3}=S_{2}+\kappa_{e}\mu_{i}^{2}I-P_{2}C_{au,i}\vartheta_{1}\dot{\hat{d}}\vartheta_{2}UD-{\left(C_{au,i}\vartheta_{1}\dot{\hat{d}}\vartheta_{2}UD\right)}^{T}P_{2}$$ (73)

then the estimate errors e_au_ and e_d_ are uniformly ultimately bounded under the estimation algorithm (68).

*Proof*. Let

$$\tilde{e}=[\begin{array}{c} e_{au}\\ e_{d} \end{array}]$$ (74)

The Lyapunov function is selected as follows:

$$V_{e}={\tilde{e}}^{T}P_{e}\tilde{e}$$ (75)

where *P*_*e*_ is a symmetric positive-definite matrix.

Based on (74) and (75), we can obtain

$$\dot{\tilde{e}}=\sum_{i=1}^{w}\left(\varphi_{i,low}+\varphi_{i,up}\right)[({\tilde{A}}_{i}+{\tilde{A}}_{s})\tilde{e}+{\tilde{B}}_{i}{\tilde{g}}_{i}-{\tilde{C}}_{i}\dot{d}]$$ (76)

where

$${\tilde{g}}_{i}=g_{i}(\hat{x},t)-g_{i}(x,t)$$ (77)

$$\left\{ \begin{array}{l} {\tilde{A}}_{i}=\left[\begin{array}{cc} M_{i} & \vartheta_{1}\dot{\hat{d}}\vartheta_{2}UD\\ -\Gamma_{2}C_{au,i}-\Gamma_{2}C_{au,i}M_{i} & -\Gamma_{2}C_{au,i}\vartheta_{1}\dot{\hat{d}}\vartheta_{2}UD \end{array}\right]\\ {\tilde{A}}_{s}=\left[\begin{array}{cc} M_{s} & 0\\ -\Gamma_{2}C_{au,i}M_{s} & 0 \end{array}\right]\\ {\tilde{B}}_{i}=\left[\begin{array}{c} \vartheta_{1}\dot{\hat{d}}\vartheta_{2}U\\ -\Gamma_{2}C_{au,i}\vartheta_{1}\dot{\hat{d}}\vartheta_{2}U \end{array}\right]\\ {\tilde{C}}_{i}=\left[\begin{array}{c} 0\\ I \end{array}\right] \end{array}\right.$$ (78)

The derivative of (75) can be written as follows:

$$\begin{array}{l} {\dot{V}}_{e}=\overset{w}{\underset{i=1}{\sum }}(\varphi_{i,low}+\varphi_{i,up})\{{\tilde{e}}^{T}[{\left({\tilde{A}}_{i}+{\tilde{A}}_{s}\right)}^{T}P_{e}+P_{e}({\tilde{A}}_{i}\\ +{\tilde{A}}_{s})]\tilde{e}+2{\tilde{e}}^{T}P_{e}{\tilde{B}}_{i}{\tilde{g}}_{i}-2{\tilde{e}}^{T}P_{e}{\tilde{C}}_{i}d]\\ \leq \overset{w}{\underset{i=1}{\sum }}(\varphi_{i,low}+\varphi_{i,up})\{{\tilde{e}}^{T}[{\left({\tilde{A}}_{i}+{\tilde{A}}_{s}\right)}^{T}P_{e}+P_{e}({\tilde{A}}_{i}+{\tilde{A}}_{s})\\ +\kappa_{e}^{-1}P_{e}{\tilde{B}}_{i}{\left(P_{e}{\tilde{B}}_{i}\right)}^{T}+\kappa_{e}\mu_{i}^{2}I]\tilde{e}-2{\tilde{e}}^{T}P_{e}{\tilde{C}}_{i}d\} \end{array}$$ (79)

Based on Lemma 1, there exists a matrix *S* = *S*^*T*^>0 such that

$$\begin{array}{l} -2{\tilde{e}}^{T}P_{e}{\tilde{C}}_{i}d\leq {\tilde{e}}^{T}S\tilde{e}+{\dot{d}}^{T}{\left(P_{e}{\tilde{C}}_{i}\right)}^{T}S^{-1}P_{e}{\tilde{C}}_{i}d\\ \leq {\tilde{e}}^{T}S\tilde{e}+d_{1}^{2}\max(\mathrm{eig}({\left(P_{e}{\tilde{C}}_{i}\right)}^{T}S^{-1}P_{e}{\tilde{C}}_{i})) \end{array}$$ (80)

Denote

$$\eta_{2}=\sum_{i=1}^{w}\left(\varphi_{i,low}+\varphi_{i,up}\right)d_{1}^{2}\max(eig({\left(P_{e}{\tilde{C}}_{i}\right)}^{T}S^{-1}P_{e}{\tilde{C}}_{i}))$$ (81)

Then,

$$\begin{array}{l} {\dot{V}}_{e}\leq \sum_{i=1}^{w}\left(\varphi_{i,low}+\varphi_{i,up}\right)\{{\tilde{e}}^{T}[{\left({\tilde{A}}_{i}+{\tilde{A}}_{s}\right)}^{T}P_{e}+P_{e}({\tilde{A}}_{i}\\ +{\tilde{A}}_{s})+S+\kappa_{e}^{-1}P_{e}{\tilde{B}}_{i}{\left(P_{e}{\tilde{B}}_{i}\right)}^{T}+\kappa_{e}\mu_{i}^{2}I]\tilde{e}+\eta_{2}\}\\ =\sum_{i=1}^{w}\left(\varphi_{i,low}+\varphi_{i,up}\right)\\ \{{\tilde{e}}^{T}\left[\begin{array}{cc} {\left({\tilde{A}}_{i}+{\tilde{A}}_{s}\right)}^{T}P_{e}+P_{e}({\tilde{A}}_{i}+{\tilde{A}}_{s})+S+\kappa_{e}\mu_{i}^{2}I & P_{e}{\tilde{B}}_{i}\\ {}* & -\kappa_{e}I \end{array}\right]\tilde{e}\\ +\eta_{2}\} \end{array}$$ (82)

Denote

$$\Xi_{i}=[\begin{array}{cc} {\left({\tilde{A}}_{i}+{\tilde{A}}_{s}\right)}^{T}P_{e}+P_{e}({\tilde{A}}_{i}+{\tilde{A}}_{s})+S+\kappa_{e}\mu_{i}^{2}I & P_{e}{\tilde{B}}_{i}\\ {}* & -\kappa_{e}I \end{array}]$$ (83)

And suppose

$$P_{e}=[\begin{array}{cc} P_{1} & 0\\ 0 & \Gamma_{2}^{-1}P_{2} \end{array}],S=[\begin{array}{cc} S_{1} & 0\\ 0 & S_{2} \end{array}].$$

Then, the following can be obtained:

$$\Xi_{i}=[\begin{array}{ccc} \Psi_{i1} & \Psi_{i2} & P_{1}\vartheta_{1}\dot{\hat{d}}\vartheta_{2}U\\ {}* & \Psi_{i3} & -P_{2}C_{au,i}\vartheta_{1}\dot{\hat{d}}\vartheta_{2}U\\ {}* & * & -\kappa_{e}I \end{array}]$$ (84)

$$\{\begin{array}{l} \Psi_{i1}={\left(M_{i}+M_{s}\right)}^{T}P_{1}+P_{1}(M_{i}+M_{s})+S_{1}+\kappa_{e}\mu_{i}^{2}I\\ \Psi_{i2}=P_{1}\vartheta_{1}\hat{\hat{d}}\vartheta_{2}UD-{\left(M_{i}+M_{s}\right)}^{T}C_{au,i}^{T}P_{2}-C_{au,i}^{T}P_{2}\\ \Psi_{i3}=S_{2}+\kappa_{e}\mu_{i}^{2}I-P_{2}C_{au,i}\vartheta_{1}\hat{\hat{d}}\vartheta_{2}UD-{\left(C_{au,i}\vartheta_{1}\hat{\hat{d}}\vartheta_{2}UD\right)}^{T}P_{2} \end{array}$$ (85)

Therefore, (82) can be rewritten as

$${\dot{V}}_{e}\leq \sum_{i=1}^{w}\left(\varphi_{i,low}+\varphi_{i,up}\right)({\tilde{e}}^{T}\Xi_{i}\tilde{e}+\eta_{2})$$ (86)

FLAD is bounded, if (70) is true, that is, every linear mode obtained by real-time calculation of the left matrix in (70) is negative, then denote *σ*_*i*_ = min(eig(−Ξ_*i*_)). Thus,

$${\dot{V}}_{e}\leq \sum_{i=1}^{w}\left(\varphi_{i,low}+\varphi_{i,up}\right)(-\sigma_{i}{\left\|\tilde{e}\right\|}^{2}+\eta_{2})$$ (87)

When ${\left\|\tilde{e}\right\|}^{2}>\sum_{i=1}^{w}\left(\varphi_{i,low}+\varphi_{i,up}\right)(\eta_{2}/\sigma_{i})$, we can obtain the following.


$${\dot{V}}_{e}<0$$ (88)

That is, error dynamics can converge to an interval

$$\tilde{e}(t){\left\|\tilde{e}(t)\right\|}^{2}\leq \sum_{i=1}^{w}\left(\varphi_{i,low}+\varphi_{i,up}\right)\max(\eta_{2}/\sigma_{i})$$ (89)

Therefore, *e*_*au*_ and *e*_*d*_ are ultimately uniformly bounded. This completes the proof of Theorem 3.

Obtain the LMI cluster through real-time calculation (70) and use Schur’s supplementary lemma, feasible solutions (*P*_1_, *P*_2_, *M*_*i*_, *M*_*s*_, *κ*_*e*_) can be obtained. Then, *N*_*i*_ and *N*_*s*_ can be determined using (65). Accordingly, observer (61) is determined.

Through the isolation process, the unknown matrix, *F*, as well as *F*_*au*_, is determined; without a loss of generality, *F*_*au*_ is the full column rank. Then, the estimation of the two sensor faults and output noise can be achieved via the following augmented observer.


$$\{\begin{array}{l} \hat{f}=[I_{2}\;0_{2\times 1}]{\left(F_{au}^{T}F_{au}\right)}^{-1}F_{au}^{T}{\hat{x}}_{f\omega }\\ \hat{\omega }=[0_{1\times 2}\;1]{\left(F_{au}^{T}F_{au}\right)}^{-1}F_{au}^{T}{\hat{x}}_{f\omega }\\ {\hat{x}}_{f\omega }=[0_{5\times 5}\;I_{5}]{\hat{x}}_{au} \end{array}$$ (90)

Obtaining valuable information about sensor faults, FLAD, and noise can aid in designing an active FTC scheme.

### 3.4. Fault compensation

The design of the nominal controller allows the variable-structure HFV to fly stably and track the altitude and velocity commands. However, when multi-sensor faults occur, the HFV will be inaccurate. Moreover, because FLAD will also affect flight performance, the HFV must be able to overcome external FLAD. Therefore, a robust variable-structure fault compensation based on the diagnosis results is essential.

When sensors fail, the actual measurement *y*(*t*) changes into *y*^*f*^(*t*) that provides wrong signals for the basic output feedback controller. Thus, *y*^*f*^(*t*) should be substituted by a compensated output *y*_*c*_(*t*) with

$$y_{c}(t)=y^{f}(t)-{\hat{x}}_{f\omega }(t)$$ (91)

The FLAD should also be counteracted to make the flight of the variable-structure HFV smooth. Hence, the variable-structure FTC laws are designed as follows:

$$\begin{array}{l} u=-\overset{w}{\underset{i=1}{\sum }}(\varphi_{i,low}+\varphi_{i,up})\{(K_{i}+\Gamma_{3}\dot{\hat{d}}\Gamma_{4})e_{yd}+{\left(B_{i}+B_{s}\right)}^{+}[v_{i}\\ +(A_{i}+A_{s})y_{d}+D\hat{d}]\} \end{array}$$ (92)

$${\dot{\nu }}_{i}=\Gamma_{1}P_{f1}(e_{yd}+{\dot{e}}_{yd})$$ (93)

$$\begin{array}{l} e_{yd}(t)=y_{c}(t)-y_{d}\\ =y^{f}(t)-{\hat{x}}_{f\omega }(t)-y_{d} \end{array}$$ (94)

*Remark 3*. The FTC laws, (91)–(94), render the HFV robust against both input disturbance and output noise. The control precision of (94) is better than that of $y_{c}(t)=y^{f}(t)-F\hat{f}(t)$ because the latter does not consider the output noise.

After introducing (92)–(94), (95) is derived as follows:

$$\begin{array}{l} {\dot{e}}_{yd}(t)=\sum_{i=1}^{w}\left(\varphi_{i,low}+\varphi_{i,up}\right)\{[A_{i}+A_{s}-(B_{i}\\ +B_{s})(K_{i}+\Gamma_{3}\dot{\hat{d}}\Gamma_{4})]e_{yd}(t)+e_{gi}-{\dot{e}}_{f\omega }(t)-De_{d}(t)\}\\ =\sum_{i=1}^{w}\left(\varphi_{i,low}+\varphi_{i,up}\right)\{[A_{i}+A_{s}-(B_{i}+B_{s})(K_{i}\\ +\Gamma_{3}\dot{\hat{d}}\Gamma_{4})]e_{yd}(t)+e_{gi}-[0_{5\times 5}\;I_{5}]{\dot{e}}_{au}(t)-De_{d}(t)\}\\ =\sum_{i=1}^{w}\left(\varphi_{i,low}+\varphi_{i,up}\right)\{[A_{i}+A_{s}-(B_{i}+B_{s})(K_{i}\\ +\Gamma_{3}\dot{\hat{d}}\Gamma_{4})]e_{yd}(t)+e_{gi}-[0_{5\times 5}\;I_{5}](M_{i}+M_{s})e_{au}(t)\\ -[0_{5\times 5}\;I_{5}]U^{*}{\tilde{g}}_{i}-([0_{5\times 5}\;I_{5}]U^{*}D+D)e_{d}(t)\} \end{array}$$ (95)

where

$$e_{f\omega }(t)={\hat{x}}_{f\omega }(t)-x_{f\omega }(t)$$ (96)

Based on Theorems 1 and 3, *e*_*gi*_ and *e*_*d*_ are known to be uniformly ultimately bounded and can be sufficiently small when suitable parameters are selected. Therefore, the effects of *e*_*gi*_ and *e*_*d*_ are neglected in the process of ensuring the stability of the FTC.

The Lyapunov function is selected as follows:

$$\begin{array}{l} V_{f}=V_{f1}+hV_{f2}\\ ≜e_{yd}^{T}P_{f1}e_{yd}+he_{au}^{T}P_{f2}e_{au} \end{array}$$ (97)

where *h* is a positive scalar, and *P*_*f*1_ and *P*_*f*2_ are two symmetric positive-definite matrices with appropriate dimensions.

Thus, it can be obtained as follows:

$$\begin{array}{l} {\dot{V}}_{f2}=\overset{w}{\underset{i=1}{\sum }}(\varphi_{i,low}+\varphi_{i,up})\{h[e_{au}^{T}(P_{f2}(M_{i}\\ +M_{s})+{\left(M_{i}+M_{s}\right)}^{T}P_{f2})e_{au}+2e_{au}^{T}P_{f2}U^{*}{\tilde{g}}_{i}]\}\\ \leq \overset{w}{\underset{i=1}{\sum }}(\varphi_{i,low}+\varphi_{i,up})\{h[e_{au}^{T}(P_{f2}(M_{i}+M_{s})\\ +{\left(M_{i}+M_{s}\right)}^{T}P_{f2}+\kappa_{f2}^{-1}P_{f2}U^{*}U^{*T}P_{f2}+\kappa_{f2}\mu_{i}^{2}I)e_{au}]\} \end{array}$$ (98)

where *κ*_*f*2_ is a positive scalar.

If (99) holds, then

$$[\begin{array}{cc} P_{f2}(M_{i}+M_{s})+{\left(M_{i}+M_{s}\right)}^{T}P_{f2}+\kappa_{f2}\mu_{i}^{2}I & P_{f2}U^{*}\\ {}* & -\kappa_{f2}I \end{array}]<0$$ (99)

then there exist some positive scalars *б*_*i*_ such that

$${\dot{V}}_{f2}\leq -\sum_{i=1}^{w}\left(\varphi_{i,low}+\varphi_{i,up}\right)(h{б}_{i}{\left\|e_{au}\right\|}^{2})$$ (100)

Thence,

$$\begin{array}{l} {\dot{V}}_{f}={\dot{V}}_{f1}+{\dot{V}}_{f2}\\ \leq \sum_{i=1}^{w}\left(\varphi_{i,low}+\varphi_{i,up}\right)\{e_{yd}^{T}[(A_{i}+A_{s}-(B_{i}+B_{s})(K_{i}\\ +\Gamma_{3}\dot{\hat{d}}\Gamma_{4}){)}^{T}P_{f1}+P_{f1}(A_{i}+A_{s}-(B_{i}+B_{s})(K_{i}+\Gamma_{3}\dot{\hat{d}}\Gamma_{4}))]e_{yd}\\ -2e_{yd}^{T}P_{f1}[\begin{array}{cc} 0_{5\times 5} & I_{5} \end{array}]U^{*}{\tilde{g}}_{i}-2e_{yd}^{T}P_{f1}[\begin{array}{cc} 0_{5\times 5} & I_{5} \end{array}](M_{i}+M_{s})e_{au}\\ -h{б}_{i}{\left\|e_{au}\right\|}^{2}\} \end{array}$$ (101)

According to Theorem 1, there exist some positive scalars *ς*_*i*_, such that (102) holds,

$$\begin{array}{l} e_{yd}^{T}\{{\left[A_{i}+A_{s}-(B_{i}+B_{s})(K_{i}+\Gamma_{3}\hat{d}\Gamma_{4})\right]}^{T}P_{f1}+P_{f1}[A_{i}+A_{s}\\ -(B_{i}+B_{s})(K_{i}+\Gamma_{3}\hat{\hat{d}}\Gamma_{4})]\}e_{yd}\leq -\varsigma_{i}{\left\|e_{yd}\right\|}^{2} \end{array}$$ (102)

By substituting (102) into (101), the following expression can be derived:

$$\begin{array}{l} {\dot{V}}_{f}\leq \sum_{i=1}^{w}\left(\varphi_{i,low}+\varphi_{i,up}\right)\{-\varsigma_{i}{\left\|e_{yd}\right\|}^{2}-h{б}_{i}{\left\|e_{au}\right\|}^{2}\\ -2e_{yd}^{T}P_{f1}[\begin{array}{cc} 0_{5\times 5} & I_{5} \end{array}]U^{*}{\tilde{g}}_{i}-2e_{yd}^{T}P_{f1}[\begin{array}{cc} 0_{5\times 5} & I_{5} \end{array}](M_{i}\\ +M_{s})e_{au}\}\\ \leq \sum_{i=1}^{w}\left(\varphi_{i,low}+\varphi_{i,up}\right)[-\varsigma_{i}{\left\|e_{yd}\right\|}^{2}-h{б}_{i}{\left\|e_{au}\right\|}^{2}\\ +2\mu_{i}\|P_{f1}U^{*}\|\|e_{yd}\|\|e_{au}\|+2\|P_{f1}(M_{i}+M_{s})\|\|e_{yd}\|\|e_{au}\|] \end{array}$$ (103)

Let

$$h\geq \frac{4{\left(\mu_{i}\|P_{f1}U^{*}\|+\|P_{f1}(M_{i}+M_{s})\|\right)}^{2}}{\varsigma_{i}{б}_{i}}$$ (104)

then

$$\begin{array}{l} {\dot{V}}_{f}\leq \sum_{i=1}^{w}\left(\varphi_{i,low}+\varphi_{i,up}\right)(-\varsigma_{i}{\left\|e_{yd}\right\|}^{2}-h{б}_{i}{\left\|e_{au}\right\|}^{2}\\ +\sqrt{h\varsigma_{i}{б}_{i}}\|e_{yd}\|\|e_{au}\|)\\ \leq \sum_{i=1}^{w}\left(\varphi_{i,low}+\varphi_{i,up}\right)[-\varsigma_{i}{\left\|e_{yd}\right\|}^{2}-h{б}_{i}{\left\|e_{au}\right\|}^{2}\\ +0.5(\varsigma_{i}{\left\|e_{yd}\right\|}^{2}+h{б}_{i}{\left\|e_{au}\right\|}^{2})]\\ \leq \sum_{i=1}^{w}\left(\varphi_{i,low}+\varphi_{i,up}\right)(-0.5\varsigma_{i}{\left\|e_{yd}\right\|}^{2}-0.5h{б}_{i}{\left\|e_{au}\right\|}^{2})\\ <0 \end{array}$$ (105)

Thus, under FTC laws (92)–(94), system (10) remains stable, and the HFV can accurately track the given command when sensor faults and disturbances exist. Therefore, efficient variable-structure flight is achieved.

The detection, isolation, and estimation of multi-sensor bias faults and the FTC design for the HFV have been completed thus far. The simulation results explain the validity of the proposed methods in Section 4.

*Remark 4*. The proposed diagnosis and FTC schemes can deal with more sensor failures. However, the definitions of the matrices (*y*_*au*,*ks*_, *C*_*au*,*ks*_, and *H*_*au*,*iks*_) in the isolation observers and that of matrix *F* in the estimation algorithm have to be modified according to the number of faulty sensors.

## 4. Numerical simulation

This section presents and discusses the simulation results of the proposed methods. Different cases, including the FDI, fault estimation, and variable-structure FTC, are considered.

Set the prerequisite variable of the type-II fuzzy system as *x*_2_(*t*); the membership functions are

$$\{\begin{array}{l} \mu_{\xi_{i1},low}(x_{2}(t)=0.2)=\exp[-{\left((x_{2}(t)+0.5)/0.44\right)}^{2}]\\ \mu_{\xi_{i1},up}(x_{2}(t)=0.2)=\exp[-{\left((x_{2}(t)+0.5)/0.36\right)}^{2}]\\ \mu_{\xi_{i1},low}(x_{2}(t)=0.8)=1-\exp[-{\left((x_{2}(t)+0.5)/0.36\right)}^{2}]\\ \mu_{\xi_{i1},up}(x_{2}(t)=0.8)=1-\exp[-{\left((x_{2}(t)+0.5)/0.44\right)}^{2}] \end{array}.$$

Define two fuzzy rules:

*Rule 1*: If *x*_2_ is about 0.2, Then: *i* = 1, {*A*_1_ *B*_1_ *C*_1_ *K*_1_ *g*_1_(*x*, *t*)}; *Rule 2*: If *x*_2_ is about 0.8, Then: *i* = 2, {*A*_2_ *B*_2_ *C*_2_ *K*_2_ *g*_2_(*x*, *t*)}.

The nonlinear terms are set as *g*_1_(*x*, *t*) = 0.05sin(*αt*) and *g*_2_(*x*, *t*) = 0.5sin(*αt*), the non-zero value in each mode is only the first element, and the remaining elements are 0. The initial and desired states are listed in Table 1.

**Table 1:** Initial and desired data.

Initial parameter	Value	Desired parameter	Value
*V*	3750	*V*_*d*_	3850
*γ*	0	*γ*_*d*_	0
*h*	33500	*h*_*d*_	34500
*α*	0	*α*_*d*_	0
*q*	0	*q*_*d*_	0

Velocity and altitude sensor faults are considered in the simulation because these two sensors are prone to failing in the HFV. Gaussian output white noise, *ω*(*t*), with a power of 1 is added to the velocity and altitude channels. For illustrating the ability of the proposed adaptive augmented observer to estimate disturbances, a disturbance with fast-varying characteristics that is, FLAD, is selected and added to the pitch rate channel. The detailed information of faults and FLAD are as follows:

$f_{v}=\{\begin{array}{l} 0,\;t<20s\\ 30m/s,\;t\geq 20s \end{array},\;f_{h}=\{\begin{array}{l} 0,\;t<25s\\ 100\sin(1.07t-2)m,\;t\geq 25s \end{array},\;f=[\begin{array}{c} f_{v}\\ f_{h} \end{array}]$, $F={[\begin{array}{ccccc} 1 & 0 & 0 & 0 & 0\\ 0 & 0 & 1 & 0 & 0 \end{array}}^{T}]$, $D={\left[\begin{array}{ccccc} 0 & 0 & 0 & 0 & \pi /60 \end{array}\right]}^{T}$, $F_{\omega }={\left[\begin{array}{ccccc} 0.1 & 0 & 0.3 & 0 & 0 \end{array}\right]}^{T}$, d(t)=0.04sin(1.05t+2.64).

The values of active variable-structure parameters, *A*_*s*_ and *B*_*s*_, are obtained as follows:

$$A_{s}=\{\begin{array}{l} 0_{5\times 5},t<20s\\ I_{5\times 5},t\geq 20s \end{array},\;B_{s}=\{\begin{array}{l} 0_{2\times 5},t<20s\\ \left[\begin{array}{ccccc} 0.5 & 0 & 0 & 0 & 0\\ 0 & 0 & 0.5 & 0 & 0 \end{array}\right],t\geq 20s \end{array}.$$.

**Fig 2:**
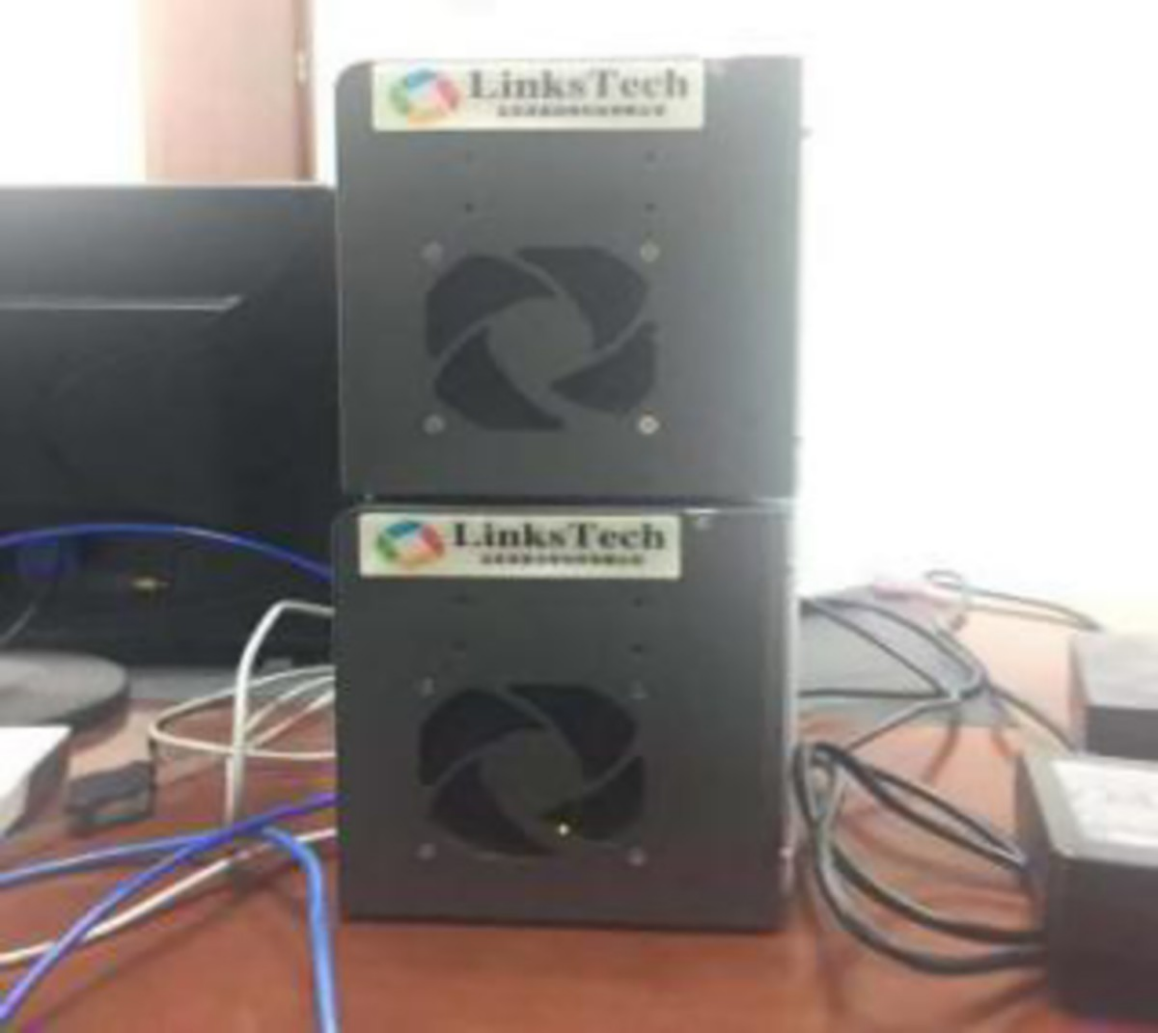
Simulation platform.

The Links-Box semi-physical platform can simulate the above real flight environment and verifying the algorithm availability, as shown in Fig 2.

### 4.1. Fault detection and isolation

**Fig 3:**
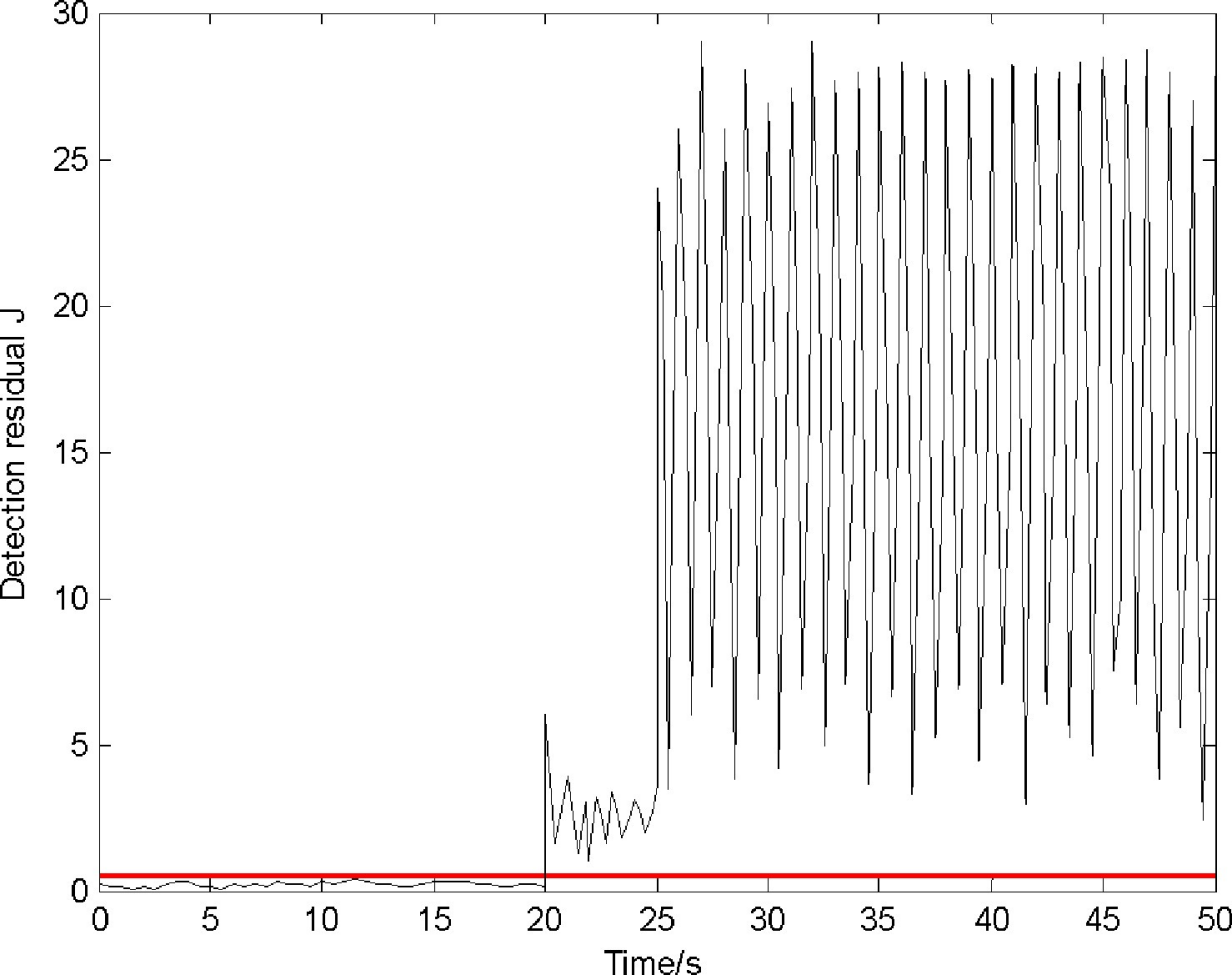
Detection residual and threshold.

**Fig 4:**
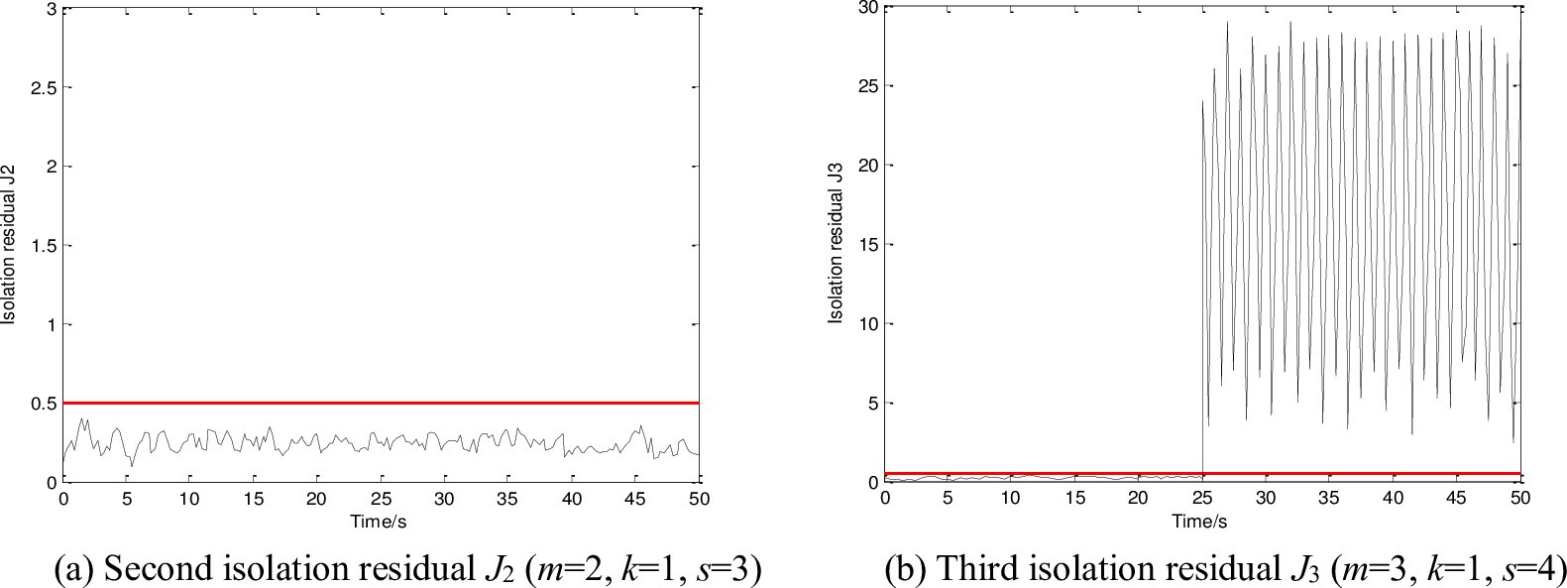
Isolation residuals and threshold. (a) Second isolation residual *J*_2_ (*m* = 2, *k* = 1, *s* = 3), (b) Third isolation residual *J*_3_ (*m* = 3, *k* = 1, *s* = 4).

Ideally, a threshold exceeding zero indicates a fault. However, because of disturbance, the threshold is no longer zero. Moreover, because the initial state of the observer (30) is set to be consistent with the original system (10), *r*(0) is zero. Therefore, *β* can be set as any constant greater than one. The maximum values of the three disturbance/noise channels are 0.05, 0.1, and 0.3; *ε* is set as 0.5. Hence, 0.5 is selected as the warning threshold to reduce false/missing reports. The FDI results are shown in Figs 3 and 4.

Fig 3 shows that the detection residual exceeds the threshold (0.5) after 20 s (i.e., after the sensor faults occur). It should be noted that the residual increases rapidly at 20 and 25 s after the velocity and altitude sensors begin to fail, respectively. Fig 4 only presents the residuals of the second and third observers of the total 10 observers. As shown in Fig 4(A), the residual is less than 0.5; in Fig 4(B), the residual starts to exceed 0.5 at 25 s. Thus, the first and third sensors (velocity and altitude sensors, respectively) are observed to have failed.

### 4.2. Fault and disturbance estimation

**Fig 5:**
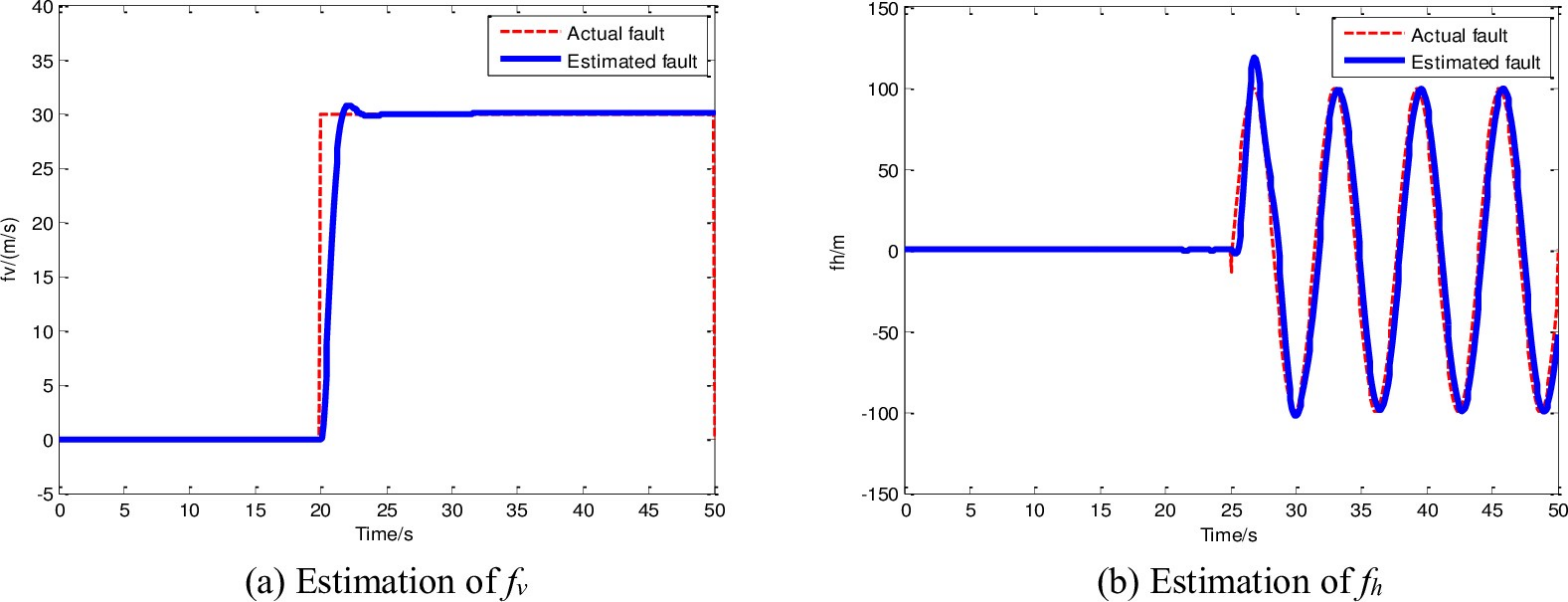
Fault estimation with the adaptive augmented observer. (a) Estimation of *f*_*v*_, (b) Estimation of *f*_*h*_.

**Fig 6:**
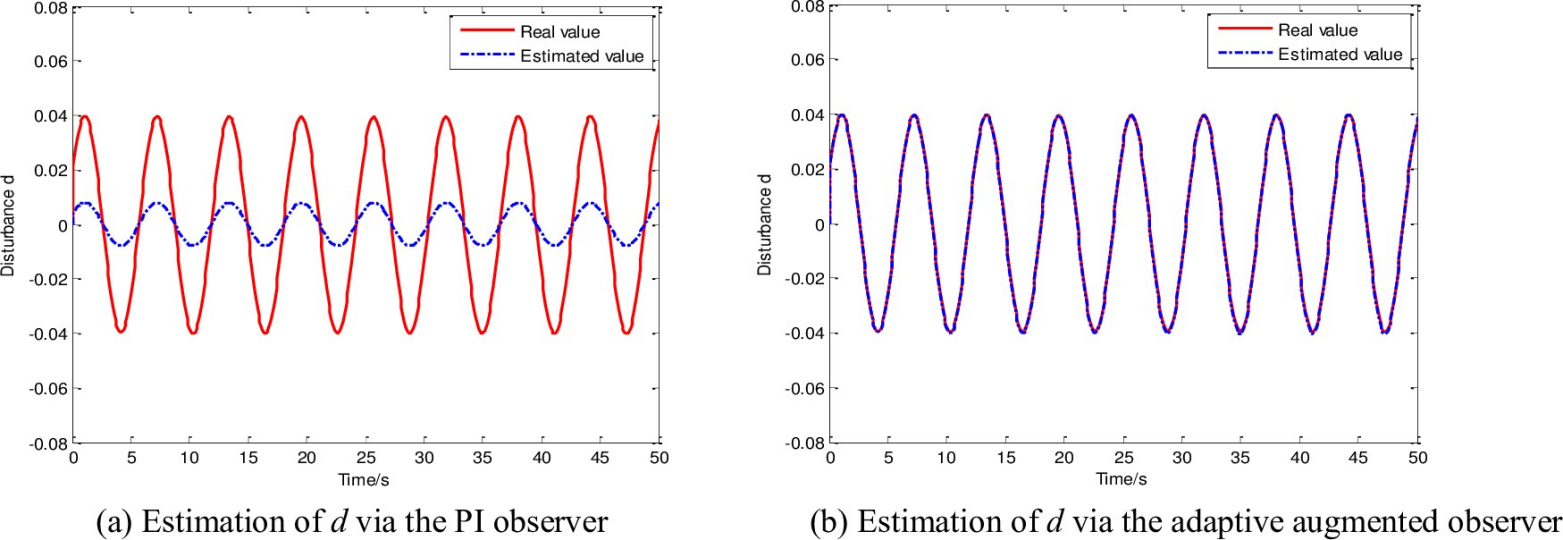
Disturbance estimation results via different observers. (a) Estimation of *d* via the PI observer, (b) Estimation of *d* via the adaptive augmented observer.

In Section 4.2, we present the estimation results of the two faulty sensors and the disturbance through the adaptive augmented observer to illustrate the effectiveness of the proposed estimation scheme. Among the two sensor faults, one is constant, and the other is time-varying. To demonstrate the superiority of the proposed observer, the disturbance estimation result obtained using the observer is compared with that obtained using the PI observer in [26]. The fault and disturbance estimation results are shown in Figs 5 and 6, respectively.

Fig 5 shows that the fault observer can flexibly estimate different types of faults in different control loops. It can stably and accurately track both the velocity constant fault ((a)) and altitude time-varying fault ((b)), create the condition for the active FTC.

Fig 6 shows the superior performance of the observer proposed in this paper when dealing with FLAD. Fig 6(A) shows the estimation result of the PI observer in [26]; evidently, it cannot satisfy the accuracy requirement. The results obtained using the observer presented in this paper are shown in Fig 6(B). The estimation of FLAD is fast and stable, and the tracking error is practically zero. So the proposed observer improves the existing method.

### 4.3. Fault compensation

**Fig 7:**
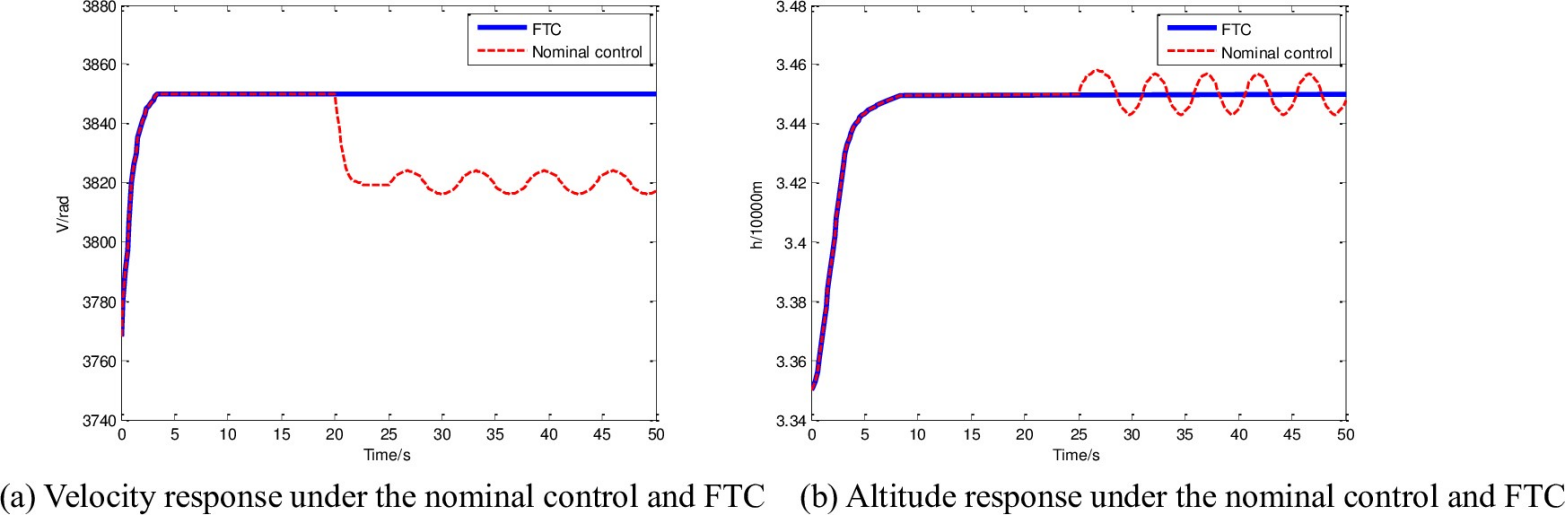
Outputs with different controllers in sensor fault case. (a) Velocity response under the nominal control and FTC, (b) Altitude response under the nominal control and FTC.

To illustrate the importance of fault compensation, this section presents the velocity and altitude curves that are obtained under the nominal control and FTC. The simulation results are shown in Fig 7.

Fig 7 shows that under fault conditions, the nominal controller cannot complete the normal velocity and altitude tracking controls. When multi-sensor faults occur, Fig 7(A) shows the velocity deviation under nominal control. The desired speed cannot be tracked at 3850 m/s, and there is a 10 m/s oscillation near the 3820 m/s deviation. Fig 7(B) indicates that the faults will also affect the tracking of the expected altitude (3450 m), and the system will oscillate approximately 16 m after 25 s. The solid line in Fig 7 indicates that the FTC can maintain the stable and accurate tracking of the two expected outputs with multi-sensor faults as well as achieve automatic fault shielding. The curves in Figs 5–7 all have slight random oscillations, the amplitude does not exceed 0.1% of the measured signal values. Due to the robustness of the system and the addition of multiple adaptive vibration suppression functions, these curves look very smooth, fully meet the engineering requirements for suppression of mechanical vibration. Our next step is to study the FTC problem of compound faults at different positions of the fuselage, the HFV flight control problem in different flight stages, and the active-passive hybrid fault repair problem.

## 5. Conclusion

In this study, the requirement for reliable control of the variable-structure HFV is addressed. When there is no fault, the proposed method directly reconstructs the algorithm with variable-structure parameters to adapt to the aerodynamic area changes of the fuselages and achieve the multimodal nonlinear control of the HFV. When multi-sensor faults occur, the FDI scheme employs improved output residuals and a threshold to complete the calibration of the failure time and locations under the FLAD conditions. Furthermore, the adaptive augmented observer with additional proportional-derivative terms accurately estimates the magnitudes of each sensor fault and fast-varying disturbance. This creates not only the conditions for the robust FTC but also solves the problem of large-amplitude disturbance that performance indicators cannot handle. Comparative experiments verify the effectiveness of the proposed method. Finally, the variable structure FTC uses the estimated information to complete the accurate tracking of velocity and altitude under multi-sensor fault conditions. This work gives the diagnosis/ compensation technology of multi-sensor faults in HFV, and has great application prospect.
